# Evaluating Therapeutic Healthcare Environmental Criteria: Architectural Designers’ Perspectives

**DOI:** 10.3390/ijerph20021540

**Published:** 2023-01-14

**Authors:** Minjung Cho

**Affiliations:** Department of Architecture, Inha University, 100 Inharo, Michuholgu, Incheon 22212, Republic of Korea; minc@inha.ac.kr

**Keywords:** therapeutic healthcare environment, healthcare facility design, architectural designer, evidence-based healthcare design practice

## Abstract

This study presents architectural designers’ perception of the importance of healthcare environmental criteria in the implementation of user-centered, therapeutic hospital design. Architectural designers with over three years of professional experience (N = 182) in South Korea were surveyed using an empirical questionnaire. The extensive interviews of 15 hospital design experts followed to interpret the survey results and discuss the barriers and suggestions for the successful delivery of therapeutic healthcare design practice. Among the 27 variables selected from the preliminary literature review, factor analyses revealed seven important therapeutic environmental criteria (i.e., management, interior design, spatial quality, service, nature and rest, ambient indoor comfort, and social program and space; χ^2^ = 1783.088, df = 300, *p* < 0.001). Analyses of variance revealed the level of importance among these criteria related to respondents’ personal and professional characteristics. Significant differences were found for the variables from the management, interior design, and spatial quality factors in relation to the respondents sex and age. For the successful delivery of therapeutic healthcare design, the design experts highlighted the implementation of evidence-based design practice that integrates local and international knowledge from various hospital users and multi-disciplinary specialists participating in the healthcare design process.

## 1. Introduction

### 1.1. Background

An extensive body of research reports a strong association between physical healthcare environmental quality and hospital users’ health benefits and well-being [1,2,3,4,5,6,7]. Scholars have investigated the elements and attributes of the hospital physical setting and their physiological and psycho-social impacts on building occupants, which contribute to the therapeutic effects of hospital environments [8,9,10,11,12]. The essential physical and psycho-social features of hospitals are studied in relationship to various hospital inhabitants’ objective and perceptive health outcomes. These environmental features are interrelated and affect healing [13,14,15]. Substantial scientific evidence has been presented based on studies of patient and healthcare staff’s post-occupancy experiences (POE), including those of other hospital inhabitants [16,17]. Such knowledge provides compelling reasons for a user-responsive, evidence-based, healthcare design approach [18,19,20]. Subsequently, a growing number of healthcare design practices have adapted the methods and processes of user-centric, evidence-based design (EBD) globally [21]. Notwithstanding the resourceful information that has been accumulated from various hospital users’ testimonials regarding healing healthcare environments, little is known about the perception of other stakeholders participating in the decision-making and design process. Especially, architectural designers’ perspectives, although playing an essential role in the implementation of hospital design, are seldom reported concerning crucial environmental criteria for the delivery of therapeutic healthcare facilities.

### 1.2. Healthcare Environmental Attributes and Their Impacts on Hospital Users Healing Experiences

This literature review examines previous research on the therapeutic healthcare environment and design in which critical physical and psycho-social environmental attributes were analyzed in relationship to the key hospital users (i.e., patients, healthcare staff, and visitors)’ objective and subjective health outcomes and healing perceptions.

#### 1.2.1. Physical Space

Elemental physical attributes are determined during the initial design phases, making them difficult to modify afterward. The underlying physical features include spatial layout, orientation, size, shape, type of room, etc. (e.g., placement and scale of public area, access and flow, inpatient/outpatient room, utility and service space, window, etc.) [9]. The complex arrangement of rooms and movement flows may cause wayfinding and accessibility problems, raising patients and visitors’ frustration and stress [22,23]. Improper spatial arrangement caused unnecessary trips and flow overlap among hospital nurses causing delayed treatment, fatigue, and unsatisfactory performance [24,25]. The layout and shape of patient rooms and corridors that enhance wayfinding, visibility, safety, access, and efficiency were associated with the healthcare staff’s satisfaction and work performance in the emergency department [26]. Regarding the size and type of room, a single-person hospital room had more advantages than a multiple-person occupancy room, in terms of its organizational cost, patient care and management, and therapeutic impacts on patients in terms of privacy, lowered stress, control, lesser infection risk, and flexibility [27]. A single-family patient room type, compared to the open-bay room type, improved hospital caregivers’ satisfaction and reduced their stress levels [28]. A room with a solid wall as opposed to a room with a movable partition provided a different sense of acoustic and visual privacy for patients in the emergency department [29]. In an experimental study, the exterior appearance of the hospital building façade influenced the participants’ judgment on the perceived quality of care and expected comfort in hospitals [30].

In recent studies, the organizational and structural rigidity of hospital spaces are pinpointed because of unusually high demands on beds, equipment, and emergency rooms owing to the global spread of infectious diseases. Flexibility and efficiency of physical spaces are emphasized to foster adaptation to unexpected emergency situations [31]; for example, the transformation of empty core, shell spaces, or unfunctional areas to emergency checkup and treatment spaces, etc. Simultaneously, the physical design of hospitals needs to provide strategies to create a buffer between wards, a division between contaminated and uncontaminated areas, and dedicated decontamination spaces for healthcare workers [32].

#### 1.2.2. Ambient Indoor Comfort

Ambient environmental attributes indicate sensory-evoking features such as lighting, noise, temperature, air quality, etc. The ambient environmental quality is emphasized, as it influences hospital users’ physiological and psychological reactions including pain, infection, stress, positive or negative moods, satisfaction, social interaction, work efficiency, etc. [11,15].

Daylight is a crucial attribute that impacts healthcare environments [33]. Patients and healthcare staff experiencing daylight reported less depression and anxiety; shorter average length of stay and improved mood, social interaction, and satisfaction as compared to their counterparts with less daylight exposure [34,35,36,37]. Noise was paid special attention, as it affected patients’ negative health-related responses in sleep disruption, pain perception, cardiovascular response, hospital stay, wound recovery, etc. [38] Unwanted noise influenced hospital nurses’ stress, annoyance, miscommunication, impaired concentration, risk of errors, etc. [39].

Thermal comfort (i.e., temperature, humidity, air quality and airflow) also impacts the medical staff’s working conditions, well-being, safety, and health [40]. Comfortable thermal conditions can assist in stabilizing patients’ moods and improving healing [41]. According to Gola et al. [42], the hospital indoor air quality is associated with various dimensions including outdoor air and microclimatic factors (temperature, relative humidity, air velocity, air change, etc.), management (heating, ventilation, and air conditioning [HVAC] systems, etc.), design (room dimensions, furniture, finishing materials, etc.), and human and medical activities (users’ presence, health status, medical activities, etc.). The indoor air quality (i.e., humidity, air flow and exchange, temperature) is critical to hospital occupants’ healing because it relates directly or indirectly to discomfort, occupational disease, and hospital-acquired infection [43].

Especially concerning infection control, recent research has emphasized innovative ventilation systems catering to different healthcare needs to achieve the desired level of human comfort and improve the defense capabilities from contaminants [31]. As a solution, the combination of improved natural ventilation, HVAC systems, personalized ventilation, and exhaust systems is underscored to enhance the adequate level of indoor air quality and prevent the air transmission of infectious viruses across the hospital [44].

#### 1.2.3. Interior Design

Interior design features (i.e., furnishing, artwork, color, lighting, materials, etc.) can improve patients’ and visitors’ positive feelings and satisfaction toward the hospital’s physical setting and expected service value and enhance the quality of healthcare workers’ work life [45,46,47].

According to Chaundhury et al. [48], the suitable arrangement, installation, and maintenance of furniture and equipment are necessary for healthcare workers’ efficient and safe handling of patients. Improper furnishing (i.e., bed, chair, toilet, etc.) can increase the potential risk of patients’ falls and fall-related injuries [49]. The interior design elements such as furniture and finishing in patient rooms were essential parameters to increase illumination and affect indoor daylight performance [50]. In an experimental study, participants responded that the way a therapist’s office was decorated influenced how the therapist’s qualifications and energy were judged [51]. The use of nature-themed visual artwork improved patients’ and visitors’ emergency department waiting experiences, by distracting patients/visitors from their concerns (i.e., increasing positive “distraction activities” [52] (p. 175). Specific thematic design types (e.g., ocean, plant, animals, etc.) and color pallets (e.g., blue-green tones and pale to mid-color ranges) were preferable for children and adolescent patients for the different parts of their hospital settings [53]. In a POE study of healthcare staff in a mental hospital, Kalantari and Snell [54] combined a color scheme, graphics, and icons as a part of design innovation strategies for wayfinding improvement. Proper illumination related to healthcare workers’ enhanced care and reduced medical errors, and it contributed to a better quality of life and job satisfaction [55,56]. The interior finish materials, including the flooring, require special caution because of the incidence of falls and fall-induced injuries and maintenance reasons [22,57]. In recent studies, innovative materials were recommended because of their durability, flexibility, and eco-friendliness. Especially, as sanitary-related issues are emphasized for the bacterial and viral load reduction on the finishing surfaces, easily cleanable and replaceable materials are recommended to promote safety and maintenance efficiency in hospitals [32].

#### 1.2.4. Nature and View

Numerous studies have highlighted the impact of nature on patients’ and hospital staff’s health outcomes, satisfaction, and well-being [9,20,58,59,60]. Natural features include a view through a window, indoor and outdoor plants and gardens, representational visual media of nature, etc. [61,62,63,64].

In Grinde et al.’s review [65] of the empirical studies on indoor and outdoor environments, visual access to nature (regardless of environment) provided a higher potential for improved health benefits such as stress reduction, mental restoration, and mood enhancement, compared with lesser access to nature. Additionally, a window view of the outside natural surroundings had positive impacts on patients’ self-reported physical and mental health recovery in a residential rehabilitation center [66]. According to Park and Mattson [64], placing indoor plants in patients’ rooms accelerated patients’ recovery while also improving satisfaction with their rooms. Hospital gardens provided stress reduction and psychological relaxation to adult patients, family members, visitors, and nurses [63,67,68,69] as well as hospitalized children [60]. The visibility and physical access to the pediatric hospital gardens were examined and design recommendations were suggested to enhance visitation and physical activities in the hospital garden concerning access, function, visibility, and amenities [70,71]. The nature-inducing visual media was also reviewed. The patients who were engaged in rehabilitation sessions with simulated natural environments via virtual reality showed higher psycho-emotional health improvement than those who participated in regular rehabilitation sessions [72].

During the COVID-19 pandemic, healthcare workers reported experiencing anxiety, depression, stress, insomnia, etc. [73,74]. To mitigate the experienced distress by medical staff, a field study was conducted to develop a recharge room with multi-sensory, immersive, and nature-inspired relaxation scenery. Healthcare workers participated in the 15-min recharge room experiment and reported a significant short-term reduction in their perceived stress and a highly enjoyable experience [75].

#### 1.2.5. Safety

Safety is one of the most emphasized parameters in the healthcare environment [76]. The hospital built-environment influences hospital occupants’ safety directly or indirectly, such as through reductions in patient anxiety, patient falls and injuries, medical errors, and hospital-acquired infections [77]. For example, a safety concern was linked to patients’ emotional affects in healthcare settings; private patient rooms enabled patients’ feeling of protection and helped the facility be perceived as a safe place [78]. In psychiatric hospital facilities, unfamiliarity was associated with discomfort and insecurity perceived by patients [79]. Hospital nurses preferred visibility of patients and coworkers, more exits, and locks in case of emergency [80].

The environmental risks contributing to patients’ falls and related injuries are inadequate flooring, poor lighting, mobility hazards, suboptimal furniture, and unsuitable signposting [81]. To prevent medical errors by hospital staff, the improvement of the physical settings can minimize various problems [48]. In the emergency department, a poor entry layout, airflow circulation problems in the treatment area, etc., were associated with security and safety problems [32].

The significance of hospital-acquired cross-infection issues requires attention, as evidence has been reported in clinical environments worldwide [10,82]. Owing to the COVID-19 pandemic, hospital-acquired infection control is highly emphasized by improving air circulation control systems as well as managing the flow of staff, patients, and visitors in the entrance, lobby, corridors, etc. [32].

#### 1.2.6. Management and Maintenance

Management, organizational process, and physical design aspects are interrelated and directly or indirectly affect healthcare workers’ emotional stress, fatigue, work performance, and job satisfaction; for example, the management and organizational decision (e.g., staff education, communication, workload, and safety concern) and architectural/interior design aspects contribute to nursing staff’s medication errors, mediated by their physical and emotional stress and dissatisfaction with their work [48].

The enhanced performance functions of the hospital facility management affect cost reduction and efficiency improvement, and further foster patients’ satisfaction with the quality and reliability of hospital services [83]. The maintenance of the physical spaces and indoor environmental systems are also important management parameters to improve the quality of healthcare facilities perceived by medical staff [84].

Monitoring the maintenance and cleaning of indoor surfaces and air circulation systems is important for effective hospital operation. For example, high-performance surface materials and flexibility in usage and spatial organization help the effective management of emergency situations. Setting up the rules to operate the HVAC systems and scheduling for inspection and maintenance are crucial because the overall level of sanitary management of a hospital facility relies on the operation strategies of the indoor surface materials and building systems [43].

Rigidity and obsolescence of spaces and systems found in several hospital facility settings cause more challenges to hospital management [43]. Therefore, from the design phase of hospital facilities, identifying the optimal solutions for efficient and sustainable management is critical for the functional, technical, and economic operations of healthcare facilities [43].

#### 1.2.7. Service and Social Support

Patients’ perception of hospital service quality affects satisfaction and intention to choose the hospital [85,86]. According to Rashid and Jusoff [87], patients rely on functional aspects such as the physical facility and equipment, and the appearance of healthcare staff when evaluating the service quality of a hospital. In addition to the tangible, functional service quality components, scholars noted other attributes such as communication, accessibility, the relationship between patients and staff, security, convenience, etc., in the assessment of the healthcare service quality [88,89].

Arneill and Devlin [90] reported that the nice and warm appearance of the physical facility and equipment in the hospital setting influences participants’ perception of the quality of care provision. Patients’ perceived appraisals of the quality of the medical staff’s care, relationships, and privacy were positively related to perceived quality [8].

Social support and the perceived quality of social relationships among patients, healthcare staff, and families promoted individuals’ health through the buffering effects against stress [91,92]. Social support through informational, emotional, and tangible aids by hospital nurses and counselors helped reduce patients’ fear and anxiety effectively during pre-operation settings [93]. Nursing staff can benefit from social support programs that promote coping skills to reduce stress and enhance a supportive work environment in hospital settings [94]. To foster social support in healthcare settings, Ulrich [95] recommended convenient service facilities for comfort, rest, and socialization (e.g., waiting rooms, overnight accommodations, accessible gardens, break rooms, etc.) for families and visitors and healthcare staff.

Communication with healthcare staff has been a critical aspect for patients and families [96]. Communication with medical staff is an important parameter of caring, as patients often want to be well-informed by and interact and discuss with medical staff [97]. According to Douglas and Douglas [98], patients responded that facilities that are not usable and accessible hindered a patient-friendly hospital atmosphere.

In the new, technology-driven healthcare environment, patients’ care and treatment services have been substituted by digital healthcare communication and information technologies, both in routine and emergency medical situations [99]. For example, technology impacted the reconfiguring of spatial demands and programming (e.g., replacing or eliminating paper-based storages, archive rooms, etc.). Owing to the pandemic, technological advances allowed for remote care of patients, making resource management more efficient and reducing contact between patients and medical staff [32].

### 1.3. Research Aim

While hospital users’ perceptions of the physical spaces and healthcare services have been rigorously studied, scarce information is obtainable from healthcare facility designers’ perspectives. This research aimed to fill this gap by investigating architectural designers’ points of view concerning various healthcare environmental criteria to enhance the quality of therapeutic healthcare environments. Particularly, this study presents an empirical examination of various physical and psycho-social environmental factors to identify significant therapeutic environmental aspects, focusing on the user-centered perspective as a pivot to healthcare design.

The research questions are as follows:

- What are the essential therapeutic environmental factors perceived by architectural designers for the successful delivery of patient-centered, therapeutic, healthcare design?
- What healthcare environmental factors are perceived most importantly by architectural designers? How do such factors relate to architects’ personal and professional characteristics?
- Why are certain factors considered more important by architectural designers? Does the priority differ between their personal and firms perspectives?
- What are the hurdles in the implementation of such therapeutic healthcare environmental factors in hospital design practice? What can be suggested to improve the quality of therapeutic healthcare environments in the hospital design process?

To answer the aforementioned research questions, this empirical study was conducted by utilizing self-reported surveys among architectural design practitioners and in-person interviews with healthcare design specialists in South Korea. The data from the architects’ survey and extensive insights were based on their professional design expertise and hospital design examples implemented in South Korea.

Architectural design professionals play a key role in the delivery of a physical-spatial healthcare setting that is relatively permanent throughout the lifecycle of a hospital. Such physical and spatial features devised by architectural designers can have a critical impact on building users’ interactions with the physical setting as well as their psycho-social experiences [8]. As exemplified in prior studies, hospital designers integrate the psycho-social dimension reflecting users’ subjective perceptions and behavioral aspects to achieve a more comprehensive delivery of environmental design [21,84]. Therefore, it is worth considering architectural designers’ critical views concerning the therapeutic hospital environmental design criteria and the hurdles to implementing them in healthcare facility designs. Addressing these issues by obtaining evaluative data and extensive insights will provide valuable discussions on what can be suggested to tackle these issues for holistic design approaches. This will ultimately promote successful design delivery and elevate the quality of the therapeutic healthcare environment.

## 2. Materials and Methods

This study conducted a survey with architectural design practitioners and interviewed architects who specialized in hospital design. Based on the relevant literature resources, a survey questionnaire was developed to examine architectural design practitioners’ perspectives on the importance of therapeutic environmental variables for the success of healthcare design delivery. Following the survey, supplementary interviews with hospital design experts were conducted to interpret and expand the findings from the survey results. Interview materials were also used to discuss how to successfully integrate the therapeutic design criteria in the design process as well as the outcome of actual hospital facilities.

### 2.1. Instrument and Variables

#### 2.1.1. Survey

A preliminary list of 27 essential therapeutic healthcare environmental variables was selected from extensive literature materials. These variables include spatio-physical and psycho-social environmental attributes emphasized during the design delivery and the post-design delivery stages. Two professional architects who were awarded for the excellency of their hospital design projects in South Korea were consulted in the selection of these preliminary variables. The architects confirmed these variables are important not only in the design delivery stage but also in the post-design delivery for the successful design and operation of the healthcare facility. The draft survey questionnaire was then developed and validated by two senior healthcare specialist architects. The draft questionnaire was then updated based on their input.

The completed survey questionnaire comprised two sections:

- Respondents’ personal and professional characteristics: sex, age, education, hospital design experiences (years), architect licensure, and field of design specialty.
- Importance of therapeutic healthcare environmental attributes: 27 variables from the relevant literature sources (Table 1). Respondents were asked to evaluate the importance of the 27 variables, considering a user-centric hospital design task. Respondents indicated the degree that they considered it important. A five-point Likert scale (1 = *least important*, 2 = *not so important*, 3 = *neutral*, 4 = *important*, and 5 = *very important*) was used to assess the level of importance of the 27 variables in the delivery of therapeutic healthcare facility design.

- Utilizing the two sections, the relationship between respondents’ personal and professional profiles and the assessment of the importance of the variables was examined. Respondents’ personal and practice profiles may impact architects’ response patterns on the level of importance of the 27 variables. All 27 variables in the survey were described in both Korean and English texts to ensure the meanings were clear. Additional visual aids (e.g., hospital photos, images, or virtual reality) were not combined because the visual representation may alter the way in which survey respondents judge the meaning of the text description [127,128]. As such, respondents’ evaluations relied on their tacit and explicit architectural knowledge developed through their professional practice experiences [129].

#### 2.1.2. Interview

The written interview method was utilized owing to the spread of COVID-19 in South Korea. The interviewees were provided with the questionnaire, which was reviewed by the two senior architects who gave feedback for the survey questionnaire. To ensure item comprehensibility, the two architects provided their insight. The interview questionnaire comprised four sections. Except for Section I—the interviewees’ personal and professional characteristics’ questions—the other three sections consisted of open-ended questions that allowed interviewees to communicate their ideas freely. In Section II, the list of the 27 therapeutic healthcare environmental variables from the survey questionnaire is used. However, rather than assessing the degree of importance of each variable, the interviewees were asked to select the most important therapeutic environmental variables and write their thoughts on the significance of such variables concerning the successful implementation of the hospital environment.

The four sections of the interview questionnaire are as follows:

- Respondents’ personal and professional characteristics: sex, age, education, hospital design experiences (years), architect licensure, and the size of the firm (number of employees).
- The most important therapeutic environmental attributes among the 27 variables for the successful project delivery and the explanation of their importance from the personal point of view as well as from firms’ perspectives: a multiple selection of the variables is permitted and the reasons for the importance of each selected variable needs to be described.
- The selection of the therapeutic environmental attributes from the 27 variables that are more or less frequently implemented in the hospital design projects and the explanation of the reasons.
- Suggesting the ways to implement the therapeutic environmental attributes holistically in the healthcare facility design for the success of healing hospital design delivery.

### 2.2. Participants

#### 2.2.1. Survey

Survey participants were recruited by emailing the contact personnel at 32 randomly selected architectural design firms in Seoul, South Korea. In the email, the intent, procedure, and confidentiality of the survey results were described. Twenty-five firms agreed to participate. The size of these firms varied, ranging from small (≤30 employees) and mid-size (31–100 employees) to large offices (>100 employees). Using their office intranet email systems, the survey questionnaire was circulated along with the written description of the survey’s goal, voluntary participation, the confidentiality of the data, and reward. A total of 191 designers voluntarily completed survey questionnaires that were returned to the author via email. Out of the 191 responses, 182 questionnaires were used for further analysis owing to significant data omission in the other nine questionnaires. All respondents had at least over three years of professional architectural practice experience (one’s graduate school years were included if the person held a professional master’s degree in architectural design), which ensured their understanding of the importance of the examined therapeutic healthcare environmental variables [130]. Based on the respondents’ validation of the abstract spatial and psychological variables, their evaluation of the importance of healing in healthcare environmental variables was examined in relation to their personal and professional characteristics.

#### 2.2.2. Interview

The interview participants were contacted by emailing the contact personnel at the nine randomly selected architectural design firms in Seoul, South Korea, in which the healthcare design department or project team is in operation. The size of the firms varied, ranging from mid-size (30–100 employees) to large offices (>100 employees), as healthcare design projects are mostly executed by large architectural design offices in South Korea. In the email, the intent, procedure, confidentiality of the interview, and reward were explained. Fifteen healthcare design specialists agreed to participate in the interview. They had over 10 years of professional practice experience and were all design managers with at least five years of healthcare design project management experience. Such experiences are considered adequate to address healthcare design specialties [131]. The interviews were conducted using an interview questionnaire that was emailed to each participant. Each participant hand-wrote or typed their answers using the provided interview questionnaire. The completed interview documents were returned to the author via email. Additionally, two healthcare design specialists among the 15 interviewees were invited via an online meeting for in-depth discussions on their recent healthcare design projects that present adaptation of healing design elements in the course of design development and outcomes. Both specialists are men and from large-size architectural offices. They hold over 10 years of experience in the healthcare design field and were in senior project management positions leading the healthcare department team at their firms.

#### 2.2.3. Ethical Considerations

This study was approved by the Inha University Institutional Review Board and conducted in accordance with the Helsinki Declaration. Informed consent was obtained from all participants.

### 2.3. Analysis

#### 2.3.1. Survey

The survey data were analyzed using SPSS v. 25 (IBM, Armonk, NY, USA).

First, a basic descriptive analysis was conducted to understand respondents’ personal and professional characteristics.

Second, a factor analysis and a reliability analysis were conducted to analyze the validity and reliability of the survey results. The therapeutic environmental factors consisted of 27 variables based on the previous literature review. A principal component analysis (PCA) was conducted to extract the model. Varimax was used for the rotation method, which assumes independence between factors. A factor loading value of 0.4 or higher was selected, and the factors with an eigenvalue of one or more were extracted [132]. The Kaiser–Meyer–Olkin (KMO) coefficient, which determines the appropriateness of the sample used in the factor category, ranges from 0 to 1. The closer the KMO is to 1, the more desirable it is. When the KMO is at least 0.5, it is considered suitable for factor analysis [133]. Bartlett’s test of sphericity was used to test the validity of the PCA results. To measure the reliability between variables and internal consistency, Cronbach’s alpha (α) coefficient was used. There is no absolute criterion for a measurement tool to be recognized for its reliability; however, it is considered reliable if α is 0.6 or more [134].

Third, to compare the level of significance of the therapeutic environmental factors and variables, a descriptive analysis was used to examine the mean score of each factor and variable.

Fourth, a two-tailed *t*-test and analysis of variance were utilized to examine the relationship between the environmental factors and respondents’ personal and professional characteristics. The two-tailed *t*-test was used to reject the hypothesis by which the mean of a dependent variable (significance of the therapeutic environmental variables) is the same among the groups in the dependent variable and displays a confidence interval for the difference between mean values among the groups in the dependent variable. Significance was set at <0.05.

#### 2.3.2. Interview

The data collected from the interviews were analyzed by scrutinizing participants’ written manuscripts. Through this process of uncovering the meaning behind the texts in the documents, the similarities and differences in the interpretations of the selected priori variables and the reasons for such perspectives were carefully examined. In addition, the interviewees’ views concerning how to improve the implementation of the various therapeutic healthcare environmental elements for hospital design in practice were highlighted.

## 3. Results

### 3.1. Survey

#### 3.1.1. Respondents’ Personal and Professional Characteristics

Respondents’ personal and professional characteristics are shown in Table 2.

#### 3.1.2. Important Therapeutic Environmental Criteria (Factors and Items)

Each respondent’s perception of the level of importance of the 27 identified therapeutic environmental variables was examined using a PCA with varimax rotation. The factor loadings after rotation in the PCA are presented in Table 3. The KMO measure was computed to verify the sampling adequacy of the analysis [135]. The KMO coefficient was 0.835, which is considered high for the PCA [134]. Bartlett’s sphericity test yielded a significant value (1783.088; *p* < 0.001), indicating that the PCA is well-suited.

The PCA resulted in seven component factors with eigenvalues over Kaiser’s criterion of one. The explanatory power of the total combined variance was 64.917%. The reliability of each factor was examined by Cronbach’s α and was greater than 0.6, indicating acceptable internal reliability among the items in each factor (Table 3).

The seven factors presented in the PCA results are as follows:

Factor one is classified as “management” (eigenvalue = 2.700, variance = 10.801, Cronbach’s α = 0.761), as it includes variables such as hygiene, safety, and upkeep and maintenance.

Factor two is classified as “interior design” (eigenvalue = 2.628, variance = 10.531, Cronbach’s α = 0.776), as it includes variables such as color composition, coordinated art and image, and furniture layout.

Factor three is classified as “spatial quality” (eigenvalue = 2.571, variance = 10.285, Cronbach’s α = 0.765), as it includes variables such as openness and visibility, location and orientation, wayfinding, accessibility, spatial privacy, and exterior appearance.

Factor four is classified as “service” (eigenvalue = 2.285, variance = 9.141, Cronbach’s α = 0.754), as it includes variables such as friendly medical staff, helpful administration staff, and family service facility.

Factor five is classified as “nature and rest” (eigenvalue = 2.085, variance = 8.339, Cronbach’s α = 0.712), as it includes variables such as outdoor view, natural daylight, indoor green space, and indoor play and rest area.

Factor six is classified as “ambient indoor comfort” (eigenvalue = 2.046, variance = 8.183, Cronbach’s α = 0.679), as it includes variables such as illumination, temperature, air quality, and indoor noise.

Factor seven is classified as “social program and space” (eigenvalue = 1.914, variance = 7.655, Cronbach’s α = 0.663), as it includes variables such as the provision of facilitating spaces to run information and communication and socio-cultural programs for hospital users.

#### 3.1.3. Priority among the Therapeutic Environmental Criteria (Factors and Items)

The level of importance of each therapeutic environmental factor and variable perceived by the respondents was compared by computing the descriptive analysis of the mean and standard deviation (SD) value of each factor and variable item (Table 4). Respondents perceived that all factors and subsequent variable items were important to effective hospital design (i.e., the mean values of all items were over 3.0). Factor one, “management,” yielded the highest importance (mean = 4.52, SD = 0.573); followed by factor six, “ambient indoor comfort” (mean = 4.26, SD = 0.501); factor four, “service” (mean = 4.15, SD = 0.606) and factor five, “nature and rest” (mean = 4.04, SD = 0.556).

Concerning the variable items, “air quality” (mean = 4.66, SD = 0.597) yielded the highest mean value, followed by “hygiene” (mean = 4.65, SD = 0.620) and “safety” (mean = 4.51, SD = 0.763), “natural daylight” (mean = 4.54, SD = 0.618), and “medical staff’s service” (mean = 4.42, SD = 0.691).

#### 3.1.4. Relationship between Important Therapeutic Environmental Criteria and Respondents’ Personal and Professional Characteristics

The three therapeutic environmental factors (i.e., management, interior design, and spatial quality) were significantly related to respondents’ personal characteristics. However, the rest of the factors were non-significantly different.

First, the management factor was significantly different according to the respondents’ sex (t = 3.228, *p* = 0.002). Women (M = 4.71, SD = 0.409) displayed higher scores in the management factor compared to men (M = 4.45, SD = 0.612; Table 5).

Second, the interior design factor was significantly different according to respondents’ age (t = 2.683, *p* = 0.048). Respondents in their 50s or older (M = 3.85, SD = 0.688) showed the highest scores in the interior design factor, followed by those in their 40s (M = 3.63, SD = 0.648), 30s (M = 3.55, SD = 0.705) or 20s (M = 3.37, SD = 0.753; Table 6).

Third, the spatial quality factor was significantly different according to respondents’ sex (t = 2.608, *p* = 0.010). Women (M = 3.91, SD = 0.391) showed higher scores in the spatial quality factor compared to men (M = 3.71, SD = 0.590; Table 7).

### 3.2. Interview

#### 3.2.1. Participants’ Personal and Professional Characteristics

Participants’ personal and professional characteristics are shown in Table 8.

#### 3.2.2. Priority in Design Implementation

Participants’ judgment of the priority of the therapeutic environmental criteria when implementing in design was asked both from the interviewees’ personal as well as their firms’ practice dimensions. Participants were then asked which environmental criteria are more/less frequently implemented in their hospital design practice. For each question, multiple answers among the given environmental criteria are possible.

**Figure 1:**
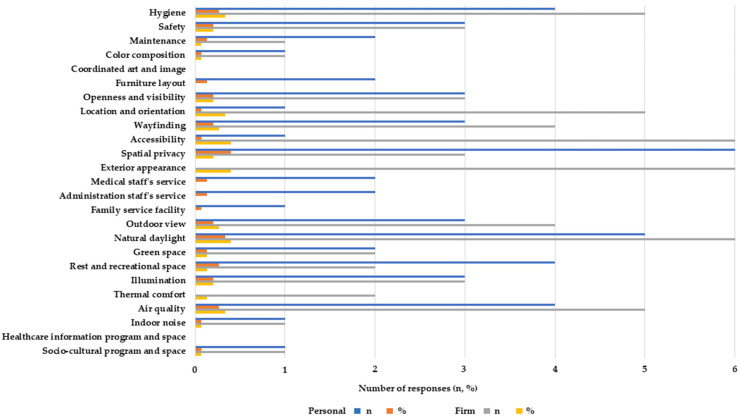
Participants’ personal priority and their firms’ priority in design implementation of the therapeutic healthcare environmental variables (Appendix A: Table A1).

Concerning participants’ personal dimensions, interviewees answered that spatial privacy (*n* = 6, 40.0%), natural daylight (*n* = 5, 33.3%), air quality (*n* = 4, 26.7%), hygiene (*n* = 4, 26.7%) and a rest and recreational space (*n* = 4, 26.7%) were among the top prior variables. Concerning the firms’ practice dimension, interviewees responded that accessibility (*n* = 6, 40.0%), exterior appearance (*n* = 6, 40.0%), natural daylight (*n* = 6, 40.0%), hygiene (*n* = 5, 33.3%), locationand orientation (*n* = 5, 33.3%), and air quality (*n* = 5, 33.3%), were critical (Figure 1).

**Figure 2:**
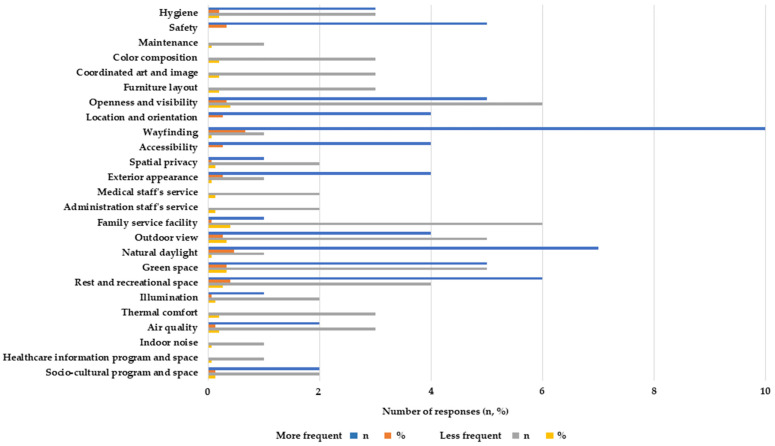
Frequency of applied variables in design implementation for therapeutic healthcare environments (Appendix A: Table A2).

Concerning more frequently (or less) implemented environmental criteria in design practice, wayfinding (*n* = 10, 66.7%) was the most common, followed by natural daylight (*n* = 7, 46.7%) and a rest and recreational space (*n* = 6, 40.0%). Contrastingly, the interviewees agreed that facilitating family service space(*n* = 6, 40.0%), creating a sense of openness and visibility (*n* = 6, 40.0%), and introducing green space (*n* = 5, 33.3%) are relatively not applied as often in their design projects (Figure 2).

#### 3.2.3. Hurdles and Suggestions in Design Practice

**Figure 3:**
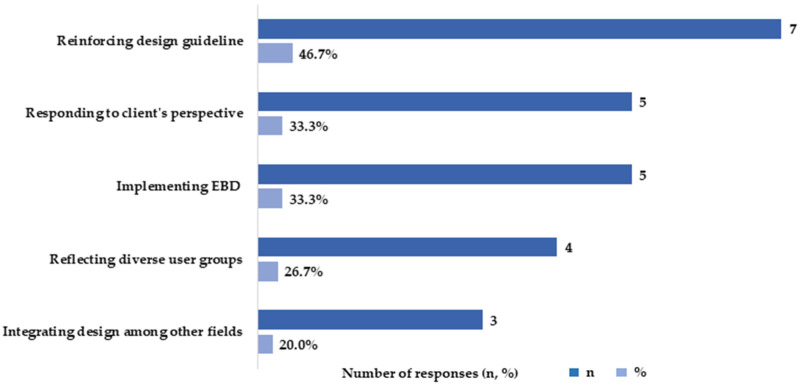
Hurdles and suggestions in healthcare design practice.

The participants addressed existing barriers and suggested ways in which to improve creating therapeutic healthcare environments in the hospital design practice (Figure 3). Participants (*n* = 7, 46.7%) strongly advocated for facilitating the design of guidelines/manuals or even imposing design restrictions for some fundamental therapeutic design elements such as the rest area, green space, natural light, access control/separation for hygienic areas, etc. For example, the provision of a mandatory rest area per a hospital’s size, or per the number of total beds, can be one of the ways to apply healing environmental criteria in the hospital.

Participants (*n* = 5, 33.3%) pinpointed that clients’ perspective is one of the primary reasons that hinders the implementation of a therapeutic healthcare environmental. Providing a healing space is often pushed out of priority from the hospital’s project budget owing to minimal interest from clients.

Participants (*n* = 5, 33.3%) strongly emphasized the significance of the evidence-based healthcare design practice approach integrating POE and case studies. According to the respondents, access to POE information is often limited in the healthcare facility precedents in South Korea. The lack of such relevant POE data on the cases of South Korean hospitals hinders the implementation of certain healing environmental items; the scarce and nonvague information creates doubts about the effects of such design elements, which makes them difficult to apply. The lack of domestic studies also challenges the ability to persuade clients and relying on overseas’ examples has limitations for both designers and clients.

Consideration of diverse hospital user groups is also emphasized by the interviewed experts (*n* = 4, 26.7%). The respondents experienced that the medical staff is the primary source of data during the user consultation process; thus, the staff’s opinion is usually adopted in the hospital’s spatial program, subsequently impacting the final design result. Since most of the clients are medical doctors or medical professors, it becomes difficult to equally reflect the diverse views of various hospital user groups.

Lastly, design integration among diverse fields in the hospital design process was highlighted by the participating experts (*n* = 3, 20.0%). Like other building types, a hospital facility requires a complex and multi-layered design process involving diverse specialty areas (i.e., architectural design; interior design; structural design; mechanical, electrical, and plumbing engineering; landscape design; accessibility checks; fire egress consulting; the Internet of things (IoT) engineering; service sector consulting, etc.). However, the coordination of such fields can be challenging, particularly when focusing on the healing aspects of hospital design, primarily owing to the limited budget allocation for such items. In addition, architectural designers are in charge of many tasks from external building design to medical space arrangement, technical solutions, permitting, etc. As such, the specificity of each expertise is limited, it becomes difficult to present a well-orchestrated healing environment.

## 4. Discussion

### 4.1. Important Therapeutic Environmental Criteria

The factor analysis presented seven environmental factors (i.e., management, spatial quality, ambient indoor comfort, interior design, service, social program and space, and nature and rest). Though the architects considered the seven factors were all important assets in pursuit of healing environments in hospital design, they prioritized the management, indoor comfort, service, and nature and rest criteria. This result highlights physical design attributes and their relationship with health.

Among the seven factors, the architects ranked the management criterion (i.e., hygiene, safety, and maintenance) most important for the hospitals’ healing environments. According to Reijula et al.’s [136] qualitative study, participants involved in the Finnish hospital design projects emphasized the building’s adaptability and durability as well as its aesthetic quality, highlighting both the facility’s design and management characteristics in the design process. Hygiene has been one of the most essential issues highlighted in the hospital environmental design. Particularly, hospital-associated infection has received special attention in evidence-based research and design because the physical built environment (e.g., room layout, sanitary equipment, furnishing, materials, etc.) reduced transmission of cross-infection in hospitals and improved hospital users’ wellness and satisfaction [118]. A safety attribute is also one of the most emphasized threads in the studies of healing healthcare environmental design [76]. Diverse spatial properties such as noise, lighting, patient room design, unit layout, and interior features (i.e., ergonomics of the furniture, equipment, and facility) were associated directly or indirectly with safety-related accidents [57]. The maintenance methods, skills, and technology have been studied and implemented to improve utilization, operational efficiency, and hospital workers’ productivity and service quality [137]. Ultimately, the goals and strategies of the maintenance function and performance are linked to hospital users’ satisfaction [83].

The ambient indoor comfort quality criterion (i.e., illumination, air quality, and thermal comfort) has received special attention in numerous publications, as indoor comfort attributes affect hospital users’ healing and well-being [10]. The architects’ perception of the significance of indoor comfort features was not exceptional, and only second to the management factor. According to Gola et al. [43], architectural design strategies can contribute to improving indoor air quality and its daily management problems and the exemplary suggestions relate to the building location, room exposure, layout design, mechanical and air handling unit system, structure, and material performances. In recent studies, indoor air quality is particularly underlined because of its direct and/or indirect relationship with hospital-acquired infection prevention and control during the COVID-19 outbreak. From the design perspective, hospital designers play a key role in providing preventive design strategies by pursuing environmental comfort, aesthetics, flexibility, and adaptability to combat the pandemic [116].

The service criterion was valued as an important therapeutic environmental asset by the architects. Service attributes include supportive service from healthcare workers and family service-related physical facilities. In previous studies, many different aspects of facility design were linked to the quality of healthcare service [18]. For example, Meng et al. [126] reported that service organization and physical facilities influenced patients’ satisfaction in Wuhan public hospitals. The physical design features such as spatial layout, visibility, and accessibility affected the perceived level of collaboration and efficient communication among healthcare staff [138]. A supportive hospital environment for family and visitors was emphasized to promote patients’ health and well-being by providing physical service infrastructure, furniture, and room equipment (e.g., visitor meeting room, family’s overnight stay facility, play area for kids, drink/tea/coffee machine for family/visitor, etc. [98]). Additionally, the design of family support facilities (i.e., location, size, and communication equipment) encouraged family participation in care by building up social networks among patients and families [139].

The positive impacts of nature on hospital users’ stress reduction, restoration, and well-being were highlighted in numerous publications [140]. Consistently, the architects agreed that providing proximity to nature and rest space is an important criterion for healing healthcare environments. Hospital design that allows access to indoor and outdoor nature promoted positive healthcare effects among hospital users [58,62,95]. For example, the window placement and orientation in the patient room toward the outdoor nature and ample natural sunlight contributed to the health benefits of patients and medical staff [66,95]. Well-designed hospital gardens relieved stress and fostered solitude, privacy, and social support for patients and hospital staff [95]. The design methods and users’ evaluation of the quality of the healing garden design were examined [63,68,141], and the strategies to promote the usage of healing gardens were suggested by improving spatial visibility, accessibility, and functionality [70]. Concerning rest space in hospitals, an attractive waiting room design influenced patients’ perceived waiting time and satisfaction with the care service [142]. A simulation study on the layout design of the hospital emergency department examined patients’ and staff’s circulation and workflow and contributed to the reduction in patient waiting time and optimization of staff allocation [143]. Becker and Parsons [18] cited Iedema et al.’s [144] research and noted that the provision of recreational facilities influenced the level of communication among patients, hospital staff, and visitors.

### 4.2. Relationship between the Important Environmental Criteria and the Respondents’ Characteristics

Of the four personal variables, respondents’ sex and age explained the differences in perceiving the importance of the therapeutic environmental criteria such as management, ambient indoor comfort, and spatial quality factors, as being more significant. Contrarily, none of the professional characteristic variables were significant enough to explain the differences in perception of the important healing environmental criteria.

The female architects’ mean scores on the level of importance of the therapeutic environmental criterion were higher than those of their male counterparts in all therapeutic environmental criteria, among which the management and spatial quality were significant. This result echoes Mourshed and Zhao’s [84] study on healthcare providers’ perspectives concerning healthcare environmental design: female healthcare staff evaluated cleanliness, ease of maintenance, and hygiene as more important than their male counterparts. Additionally, other studies reported that female healthcare providers often presented more precautious behavioral performance regarding hospital hygienic issues (i.e., hand hygiene for hospital-acquired infections [145,146]). Mourshed and Zhao [84] explained the reason for this by citing Velle’s [147] research, in which women showed greater sensitivity and physiologic responsiveness to environmental stimuli as compared to male respondents.

The female architects were more perceptive about the spatial quality criterion (i.e., wayfinding, accessibility, openness and visibility, location and orientation, spatial privacy, and exterior appearance) compared to their male counterparts. Female architects’ higher awareness of the significance of spatial quality can be further linked to the studies on women architects’ perceptions and modes of their design practices. According to Ahrentzen and Anthony [148], who cited Franck’s [149] research, female architects present a tendency to connect with others and the world as well as valuing everyday life and experiences; a desire for inclusiveness; a responsibility for others’ needs and an acceptance toward subjectivity, complexity, and flexibility. According to Ahrentzen and Anthony [148], women do not design differently from men. Rather, they are more sensitive to human needs and willing to do more to attend to clients. Although certain studies on female architects’ characteristics toward their architectural practice and relationship with clients may have methodological limitations, including sampling, the aforementioned differences between the perceptions of female and male architects may have been further associated with the socially constructed, stereotypical projection about women and men [148].

The architects’ age presented a significant difference among the age groups with their perception of the ambient indoor design criterion (i.e., color composition, coordinated art and image, and furniture layout). Older adults had higher mean scores compared to their younger counterparts. The order of the mean score from the highest to the lowest corresponded to the oldest to the youngest age groups. According to Dalke et al. [100], the improved visual environment provided enhanced patients’ recovery, and staff’s work productivity, and ultimately promoted a sense of well-being thorough the appropriate color scheme, display of certain types of artworks, adequate material and furniture layout, proper illumination, etc. It can be speculated that the architects in the older (vs. younger) age group were more conscious about the effects of the interior design criteria as a key to creating the overall ambiance of hospitals [9].

Among the professional characteristics, no variables explained the differences in the significance of therapeutic healthcare environmental factors. This result may require further examination to shed light on the relationship between architects’ professional experiences and the perspectives on the healing effects of healthcare environmental design.

### 4.3. Implementation in Healthcare Design Practice

#### 4.3.1. Personal vs. Firms’ Professional Dimension

Concerning the healthcare design experts’ viewpoints at the personal level, natural daylight, air quality, privacy, and rest area were considered critical to enhancing healing experiences in hospitals. The experts prioritized the quality of the ambient hospital environment reinforced by the introduction of ample natural daylight and effective air circulation. Furthermore, psychological attributes such as the protection of personal privacy and the provision of a place for mental recess via a tranquil and reserved atmosphere were highlighted. One design expert stated,

“*Facilitating the good quality daylight and air flow are perhaps, the most fundamental norms in the design of healthy hospital environment. Introducing natural daylight and allowing natural ventilation as well as maintaining good indoor air quality, are essential in the design of inpatient rooms and corridors. Such design principles are also significant in outpatient areas (i.e., lobby, hall, lounge, cafeteria, etc.). Hospital facilities with good quality natural daylight and air movement affect not only building occupants’ physiological health but also their psycho-emotional wellness*.”

Besides ambient environmental elements, creating a psychologically healing atmosphere in hospitals was underlined by providing private, reserved, and tranquil spaces in patient areas. The spatial aid with screen, noise reduction, and absorption materials, and rest area with seats and proximity to an outdoor view were emphasized as fundamental steps to create a healing atmosphere in inpatient areas. Contrastingly, the hospital design experts responded differently concerning their firms’ perspective, underscoring that wayfinding, exterior appearance, space layout and orientation, and circulation distribution were prioritized. Two design experts stated,

“*In my personal view, natural elements and ambient atmosphere are primary for healing environments. However, in my firm’s sphere, physical and spatial properties become priority because spatial organization of rooms and equipment affects the overall design configuration as well as management performance and cost*.”

“*Functional layout of rooms and equipment is primal in my firms’ design approach, for the physical built environment impact on the long-term efficiency in usage, operation, and maintenance of buildings. Moreover, the physical design attributes affect the overall building aesthetics and for the most, impact on the sustainability of the hospital building*.”

According to design experts, the aesthetic style of the exterior and interior design appearances is often emphasized by clients. One design expert stated,

“*As healthcare service becomes a more competitive industry, good quality physical design is emphasized more importantly as means to establish trustful and reputable image of hospitals*.”

The design experts valued the appealing and modernized impression of hospital buildings because the appearance of internal and external areas and the subsequent healing through welcoming, engaging, and tranquil images ultimately drew positive responses from occupants concerning the hospital organization’s overall healthcare service setting [98,150].

#### 4.3.2. More vs. Less Frequent Implementation

In the hospital design implementation phase, the healthcare design experts pursue to accommodate the significant environmental criteria perceived by the design project team in addition to clients’ requests. Particularly, several experts mentioned wayfinding as the most frequently emphasized attribute, especially for the design of common areas (i.e., entrance lobby, main circulation, rest lounge, cafeteria, etc.). Natural daylight was focused on most frequently. Designers achieve wayfinding and natural daylight conditions by introducing key landmark design items such as an atrium, hospital street, light-well (e.g., air shaft, sky-well, unroofed internal or external area, etc.), skylight, etc. Providing a rest area was also highlighted in the design of common spaces—both in outpatient and inpatient areas.

Contrarily, the design specialists agreed that facilitating family service spaces became more challenging owing to the recent COVID-19 pandemic and the need to prevent cross-contamination in hospitals. One design expert stated,

“*In the past, the motto of family-oriented healthcare service led to facilitation of family service spaces such as a family lounge, caretaker’s bed, rest area for families and visitors, etc. Since the spread of severe acute respiratory syndrome and COVID-19, family spaces received lesser attention, but instead moved toward non-caretaker healthcare service approach. Especially since the COVID-19 pandemic, families and visitors’ access is restricted and service spaces for family and visitors are getting eliminated in the hospital*.”

The expert addressed that creating a sense of openness physically and visually is challenging to achieve because space is often limited. Introducing more daylight and increasing glazed window areas is less cost-effective owing to the tight project budget. Providing sufficient green space was also considered difficult because of the limited plot size and interior space. One design expert stated,

“*In many urban hospital projects, creating a sense of openness and providing sufficient green space are restricted. The complex list of a space program often requires more rooms for patients and medical services, compared to the available plot area of the project site. To offer green space, my project team used a roof-top garden. We throve to provide a separate green zone for inpatients from outpatients and visitors because of COVID-19. However, in many cases, a roof-top garden or an available lot for garden is not available owing to the complex requirement of rooms and equipment*.”

### 4.4. Barriers and Suggestions in Design Practice

The experts addressed various barriers hindering hospitals’ therapeutic environmental and suggested practical and political recommendations to overcome such hurdles in the healthcare facility design.

#### 4.4.1. Reinforcing Design Guideline

The design experts emphasized raising the bar to implement therapeutic design by reinforcing design guidelines, policies, or restrictions in the hospital design project. According to the experts, although healthcare service policies and standards are consistently updated, the policy or regulatory attention to the facility design of hospitals is relatively narrow-scoped. For example, the South Korean healthcare accreditation system does not assess the facility and environmental quality criteria as thoroughly as other auditory dimensions such as service delivery, patient care and safety, infection control and prevention, and medication management systems [151]. Two design experts stated,

“*For instance, provision of a mandatory rest area per a hospital’s square area, or per the number of bed sits can be one of the design rules to improve implementation of the healing environment in hospitals*.”

“*For an effective design input, I recommend to revise the present healthcare service accreditation system to assess the quality of healing environmental design in hospital. In most cases, for the user-oriented operation and management of the hospital’s healthcare service, the South Korean Ministry of Health and Welfare runs healthcare accreditation system to monitor patient safety and hospital’s service quality. In most cases, larger hospitals or specialized hospital programs participate in the accreditation, whereas smaller or rural healthcare facilities do not. Under the current healthcare accreditation system, the facility’s spatial and physical environmental quality is a less concerned criteria*.”

#### 4.4.2. Clients’ Perspective

The design specialists pinpointed that clients play an essential role in the process of providing a successful therapeutic healthcare environment. A project design team needs to expand clients’ philosophical approach toward creating a healing hospital setting holistically, which can be delivered through a good quality physical facility as well as the organization’s healthcare service [3]. The experts emphasized that rather than relying on the low-price-based design bidding system practiced in several healthcare design projects in South Korea, it is urgent to establish the necessary standards or guidance to assure the physical design quality. However, the costs of this are often disregarded or underestimated in the project budget planning. One design expert stated,

“*Due to the changes in healthcare service policies and regulatory issues, the design construction budget and time are often fluctuating to reflect such changes in the design process. In many cases, clients concern more on the issues of functional requirement in facility programming and building operation but are less considerate on the aspects of the healing effects that can be achieved through the careful orchestration of the physical and socio-emotional design criteria for building users*.”

#### 4.4.3. EBD Practice

The design experts emphasized the necessity and importance of the EBD approach in the design practice of hospitals. According to the specialists, empirical POE information in the healthcare design research field is rare and often difficult to access in South Korea. The experts commented that the lack of relevant POE data, especially based on the local information, is due to the internal policy of a hospital organization against the exposure of the shortcomings of a hospital’s resources, doubts about the effectiveness of POE, economic reasons, etc. Nevertheless, the specialists addressed that the POE provides useful testimonials in the decision-making process; for example, in the remodeling projects of the hospital’s internal and external areas, which takes place frequently in South Korea. Owing to the lack of local research data, hospital design relies on overseas’ information. In addition, during the consultation with clients, designers often experienced using data from other building types such as nursing home facilities, workspaces, etc. The scarcity of domestic POE data becomes a hurdle to convincing the clients and drawing an aggregable decision. One design expert stated,

“*In many cases, clients and users are healthcare professionals. They are very specific about their needs and think they know a lot more than architects in hospital design. Because of this low credibility on architects, we are often struggled to persuade clients to draw a better design solution*.”

Access to EBD data and the EBD practice permit specialized information and skillsets catered to a higher level of design performance. By offering knowledge and reasonable benchmarks on various hospital settings, design quality can be improved, and clients can be convinced through a suitable comparison [129]. However, according to the experts, many architects often do not know what sources are available and where to find them because of the disclosure of information from hospitals. Even if they find relevant information, it is often from overseas’ resources that do not fit domestic situations.

#### 4.4.4. Consideration of Diverse User Groups

The design experts commented that medical staff is often a primary source of data in the design consultation when analyzing and facilitating a hospital’s spatial program; although, there are multi-level users involved in the design of hospital projects. The design experts mentioned that they were often faced with medical healthcare practitioners and/or medical university professors during the design process. As such, it was difficult to equally reflect the needs and desires of other users such as patients, families, visitors, etc., and the healthcare staff’s viewpoints impact the overall hospital design outcome. For example, in the outpatient department, doctors’ treatment rooms are usually located on the outskirts of a building and thus have an outdoor window view and daylight. The experts highlighted that it is essential to increase opportunities to consult with various hospital users including patients, nurses, administrative technicians, etc. One design expert stated,

“*Users are knowledgeable of their needs. Particularly, the clients, mostly, healthcare professionals have very specific desires. However, the access to information on other facets of healthcare users are extremely limited*.”

Therefore, considering the complexity of hospital users and conflicts from diverse healthcare user groups, it is necessary to work together toward a common goal by responding to specific knowledge and desires from various hospital users [129]. As such, collaborative design efforts from intermediate design groups (e.g., clients, designers, researchers, etc.) to end-user groups (e.g., patients, staffs, visitors, and all other users) are necessary for successful therapeutic hospital design output [152].

#### 4.4.5. Design Integration

The design experts viewed that healthcare facilities demand a complex design process involving diverse specialties from various fields (i.e., architectural design, interior design, mechanical, electrical, and plumbing engineering, landscape design, IoT, etc.). However, the experts agreed that the coordination of such associative fields is challenging.

For example, spatial programming, planning for a future space change, technical knowledge and design skills understanding medical equipment and work processes, quality checks and control for legal and technical concerns are associated with not only hospital facility design but also medical issues, permit documentation preparation and permitting, construction administration, etc. One design expert stated,

“*In the project design team, architects are responsible and in charge of almost everything from the exterior building design to medical space arrangement, mechanical electrical and plumbing technical solutions, document permitting, design supervision during construction, etc*.”

As a downfall, a specialty in each expertise may be lacking, making it difficult to present a well-coordinated healing environment in the range of expected project costs. As such, a team-based approach that utilizes specific skills and knowledge is highly recommended. Its advantage is to proceed to a design phase via a higher level of specific and professional understanding of the function and performance involved in the hospital facility design. Such expert-based design coordination can help to draw more reasonable and rational design alternatives when facing various problems in the design phase [129].

### 4.5. Design Examples

To conclude the implications on healthcare design practice, two hospital design examples are case-studied, in which the knowledge of the significant healing design criteria is conveyed in the design outcome. These examples demonstrate how architects’ approaches to healing design are reflected in the actual design results, any obstacles, and what possible solutions can be sought to deliver the design team’s promises on therapeutic hospital place-making.

**Figure 4:**
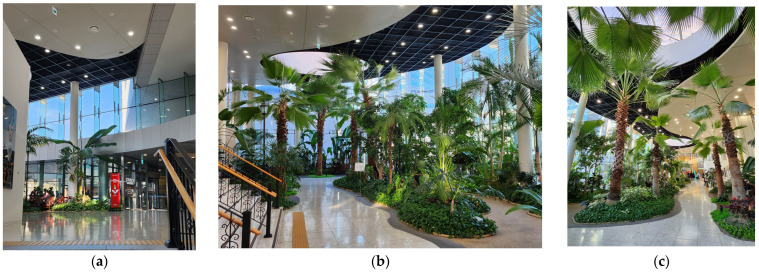
(**a**) The lounge is located in-between two buildings and connects them- the medical research lab building and a new hospital addition; (**b**) The indoor lounge includes a rest area with plants, undulating walkways, and benches; (**c**) Daylight is introduced through the glazed curtain walls and the overhead skylight in the ceiling) (Source: photos by author; The lounge was designed by Gansam Co., Ltd.).

The first example is the entrance lounge design at the Shinchon Y. S. Hospital in Seoul, South Korea, designed by Gansam Co., Ltd. (Figure 4). This project was initiated to remodel the external space between an existing medical lab building and a newly built hospital into a healing atrium space. The purpose of the design was to provide an optimal resting space that is not affected by the season and the external environment and that benefits various users from inpatients, outpatients, visitors, and hospital staff to lab users (Figure 4a).

The original space was relatively narrow and an outdoor area between two buildings and was used as a passageway with no landscaping. By turning this space into an indoor space with a natural garden and a rest area with indoor trails, the new space changed the way people use the existing hospital entrance space. Since the completion of its constriction in 2016, this lounge has intrigued not only hospital users but also passers-by to take a walk or rest, regardless of the season or weather conditions.

According to the interviewed project architect, the most emphasized design criteria were to simulate natural greenery by planting real trees from the Southern part of South Korea, introducing natural daylight and a rest space and creating a sense of openness (Figure 4b). This has also helped make the hospital entrance more appealing and promoted wayfinding as a focal point in the midst of the surrounding larger hospital complex. The concept of the lounge design was influenced by various overseas and domestic EBD information on the impact of nature on health [153,154].

“*It could have been an abandoned exterior space between the buildings, but I think this lounge design is a pinpoint at which the client’s consumer-oriented value and the architect’s design orientation are aligned. Usually, persuasion of medical clients is not an easy process because functionality, efficiency, and saving construction costs are the clients’ primary interests. But, in this case, a consensus was found between the client and the design team. The clients’ understanding of the healing space was very sophisticated. I think the most fundamental part in creating a healing environment comes through the process of finding a common point between the client’s and designer’s values*.”

However, the project architect wished to have access to POE data on the healing effects of the lounge design for building users. The project architect stated,

“*We have not found any research information from the hospital’s own investigation or from any academic research institute on this lounge design. Either such data does not exist, or even if it does, access to the data, especially if done from the hospital, would be difficult to obtain. Typically, hospitals restrict disclosure of any kind of user related data, even when such information is not relevant to their medical record*.”

Concerning design guidelines, the Korea Institute for Healthcare Accreditation provides a rough evaluation standard related to the hospital facility [155]. The architectural guideline studies for healthcare design facilities were released by the Ministry of Healthcare and Welfare [156]. These guidelines provide a broad perspective for safe and high-quality medical services by listing the standards for safety management of facility environments, facility systems, complementary hazardous materials (e.g., medical wastes, chemicals), convenience and safety facilities. As such, these guidelines are insufficient to provide the physical and psycho-social environmental aspects to convey healing hospital design [157]. The project architect mentioned,

“*Since most hospitals do not have their own facility design guidelines concerning healing environments, design guidelines are mostly tailored to the specific hospital, which are suggested by the project architects via consultation with healthcare staff during the hospital design process. This was an exemplary case in which the clients’ advocation and hospital users’ satisfaction (although not systematically researched) for the indoor healing garden were high, and the lesson from this example will allow us to pursue a similar or an upgraded indoor atrium space in other hospitals*.”

**Figure 5:**
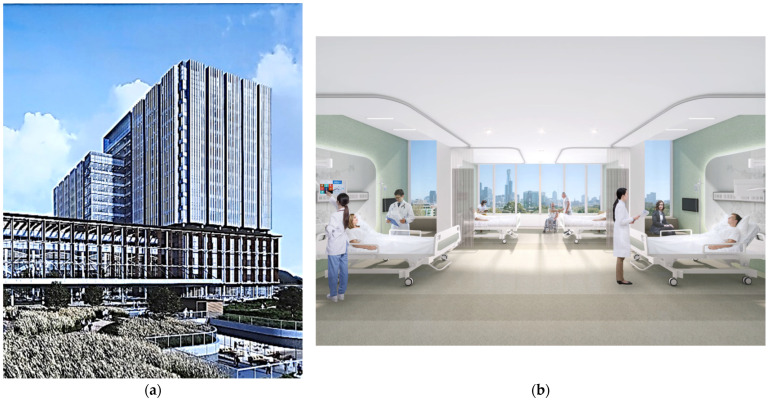
(**a**) The lower section of the hospital includes an outpatient department, central medical labs, and administrative department. The upper tower is dedicated to an inpatient department. Unlike the typical hospitals, the overall shape of the tower is not linear but rectangular to enhance the daylight condition in the inpatient wards and to reduce the corridor length traveled by nursing staff; (**b**) The design of the four-person inpatient unit type is devised to improve the daylight, view, and privacy for each patient by an extensive exterior glazed window, an alcove-shaped room, and bed arrangement. (Source: (**a**) photo by author taken from the construction site, (**b**) image from Samoo Architects & Engineers; The original competition-winning design was proposed by Samoo Architects & Engineers).

The second example is the design of inpatient wards at the Songdo Y. S. Hospital in Incheon, South Korea (Figure 5). This hospital is based on a competition-winning design proposal by the Samoo Architects & Engineers and the construction started in 2022.

South Korean urban hospitals often have outpatient and inpatient zones stacked vertically in a single building structure owing to the limited land in the city. In this hospital, the lower floors are dedicated to an outpatient department, central examination labs, administrative offices, and socio-cultural supportive spaces. The upper tower floors are composed of inpatient wards (Figure 5a).

Out of the numerous design dedications sought in this hospital proposal, the project architect emphasized the inpatient ward design. In tune with the new South Korean medical law in 2017, the inpatient ward unit design is based on a four-person room [158]. According to the project designer, the healing design concept is to secure privacy and enhance comfort via natural daylight and view. The design team researched the EBD literature on patients’ satisfaction and preference for the size, distance, and location of the patient rooms, beds, and room arrangement [159,160]. In addition, the interviews with nurses on patients’ stress and discomfort in the typical inpatient unit design were administered by the design team when initiating the design project. To provide improved privacy and daylight condition for each patient’s bed location, the design team expanded the typical unit module of 6.6 m × 9.6 m to an 8.4 m × 8.4 m module, suggested the outer periphery shape of the unit to be an alcove type, undulating form, and used extensive glazed windows. The layout of each bed allows for a direct window view, daylight, and increased patients privacy (Figure 5b). According to the interviewed project architect:

“*As a trade-off for the wider and expanded unit area, we proposed a more efficient parking layout module which can reduce dead space in the basement parking floors. This idea convinced the client because the improved inpatient ward design could improve competitiveness with similar hospitals*.”

As another effort to enhance the inpatient ward unit’s natural daylight, the design team proposed a tower with a square-shaped floor plan instead of a typical, linear floor plan. The square floor plan can allow direct contact with the outside air in four directions, which will increase the wards’ receipt of daylight from the outer periphery (Figure 5a).

“*We collaborated with an overseas design firm that conducts behavior simulation design through data algorithms. During the design process, our teams were able to create a scenario based on nurses’ daily routines from the design team’s observational studies and direct interviews with nurses because such data were not available from the hospital. By comparing the experimental results of the nurses’ simulated movement distance according to the daily routine scenario in the square vs. linear floorplan, we confirmed that the square floorplan shape reduced the nurse’s movement distance by approximately 38%. With the help of this simulation, we refined the floorplan and adjusted orientation of the overall building so that there are no wards facing due direct north. Furthermore, this data-based design collaboration resulted in higher praise from the medical clients and eventually won the competition via our unique design challenge*.”

In this example, a new multi-patient unit design was developed based on the initial research efforts by the design team’s EBD data search on inpatient satisfaction and preference with the multi-person units. Furthermore, the unit design was transformed and confirmed through the data simulation via collaborative study among the design fields. As with the completion of the hospital in a few years, the design architect wishes to investigate the impacts of the design on users such as patients’ and nurses’ stress, fatigue, satisfaction, etc. with the patient ward unit design and floorplan layout. Through this POE, the project architects expect to obtain the design improvement points which can be reflected in the follow-up attempts for the healing hospital design.

## 5. Conclusions

Designing a physical hospital environment includes a holistic approach, creating an aesthetically pleasing and functionally efficient physical setting that is responsive to human perception and behaviors [84]. In addition, healthcare facility design reflects the socio-cultural values oriented by a specific healthcare organization [161].

In the design stage, architectural designers play a fundamental role in shaping the healing hospital environment; the layout and usage of the hospital’s indoor and outdoor spaces are configured, and the spatial ambiance evolves as a result. After completion, the physical structure, primary usage, and spatial atmosphere remain relatively permanent [9] and, together, make an immense impact on hospital users’ experiences. Subsequent adjustments at a later stage can be challenging particularly considering “the multi-disciplinary nature” of healthcare facilities involving various participants and stakeholders [84]. The architect’s role is essential not only in the design stage but also in the long-term of a building’s lifecycle [162,163]. Considering their critical position in the creation of healthcare environments, considering architects’ viewpoints concerning the important therapeutic healthcare environmental criteria is an essential step to investigate a user-centric healthcare design approach and outcome.

The findings of this study highlight that architects are not only concerned with the physical place-making but also focus on the management criterion in creating healing healthcare environments. According to healthcare design specialists, architects scrutinize the issues pertaining to the post-occupancy stage during the planning stage of a building and thrive to reflect these problems in their design outcomes. Another significant finding reveals that architects highlight the psycho-social facets of the healthcare environment concerning hospital users. In particular, the results showcase that the service criteria as well as the nature and rest factors are emphasized by the architects. In many previous studies, the quality of the psycho-social setting of hospitals was linked to the perceived quality of the performance and delivery of healthcare service. This finding provides an insight into architects’ awareness of the strength of the human service factors in healing healthcare place-making and the design’s orientation to accentuate the human service delivery in the hospital.

In addition, this study sheds light on the significance of the nature and rest aspects in making a therapeutic hospital environment. Much evidence has noted the value of nature in relation to human health and well-being. The architects also agreed on the power of nature and rest in the design of healing hospitals. Therapy gardens, rest areas on the roof-top in urban hospitals, nature-themed art features in the hospital lobby and corridors, etc., exemplify the value of nature as an important asset for hospital users’ healing.

Finally, the concept of humanized healing hospitals was underlined, especially by female architects and older age designers. This finding may support previous studies, in which female architects present a tendency to approach their design practices with sensitivity, comprehensiveness, and inclusiveness based on the understanding of and empathy for human needs. The finding from this research may also recall a previous study, in which personal characteristics such as age and sex were related to the health perception, behaviors, and activities regarding a sense of well-being among South Korean adults and elders [164,165].

Methodological and practical implications of this research highlight the human-centered therapeutic medical facility design as follows:

- Avoiding obsolescence and rigidity to allow for flexible spatial reconfiguration for future expansion and particularly for effective infection control and management.
- Fostering socio-cultural service functions in healthcare facility design by integrating art, new technology, and the organization’s healthcare design and service philosophy.
- Suggesting a new design guidance or accreditation system that promotes the implementation of therapeutic healthcare design; for example, regulating a minimum rest space area per bedsit, or by a certain percentage of patient and staff areas.
- Promoting a multi-disciplinary and integrated healthcare design practice, based on scientific research data. Validated design strategies through case studies and evidence-based evaluation in domestic empirical hospital settings have not been sufficiently linked to healthcare design practices. Through integrated research and design practice, decision-making in the design and management phases can be more fluent, leading to successful healthcare design delivery.

The limitations of this study and directions for future research are as follows. In writing a future survey or conducting interviews on therapeutic hospital design elements, a questionnaire design needs to be refined in terms of validating the variables in the examination. As design is a visual field, this study’s text-based questionnaire could not fully validate the variables in question. Along with the text-oriented questionnaire, visual 2D, 3D, or real-time virtual reality representational materials can be integrated into the research design to help validation. This may convey a more concrete projection of the variables, but only if the visual information is situated in a proper healthcare context. Measuring the level of importance through the variable comparisons may be another way to refine the questionnaire design. This can help respondents precisely weigh and judge the significance of the variables. In addition, the respondents’ profiles can be measured more carefully in future research. Not only architects’ healthcare design practices but also their personal experiences with healthcare (e.g., hospitalization or visitation) may lead to different response patterns to judging the importance of therapeutic environmental elements.

In future research, more diverse and refined case studies need to be conducted. As this study aimed to identify how healing environmental elements are perceived and implemented by healthcare designers in hospital design, profound discussion on what interrupts the design delivery of healing architecture and how to improve the healing quality of the actual hospital design outcomes is necessary. Information on healthcare users’ behavioral and psychological responses to medical spaces and facilities is rarely transparent, hindering the connection between EBD research and practice. Rigorous case study investigations seeking data from diverse users’ empirical experiences in relation to actual hospital environments in various sizes and locations across South Korea as well as other regions are essential. Therefore, future researchers should endeavor to validate and expand the findings from this study through refined methodologies and further case studies.

## Figures and Tables

**Table 1:** Therapeutic healthcare environmental variables and relevant literature sources.

Variables	Citation(s)
Illumination	[55,100,101]
Air quality	[42,102,103]
Natural daylight	[34,100,101,104]
Thermal comfort	[105,106,107]
Noise	[38,39,108]
Ventilation	[107,109,110]
View	[65,66,111]
Plants and gardens	[9,58,62,63,64]
Rest and recreational space	[98,112]
Color	[62,100,104]
Art image	[113,114]
Furniture	[115]
Maintenance	[83,84]
Safety	[57,76]
Hygiene	[82,116,117,118]
Spatial access	[22,119,120]
Wayfinding	[23,121]
Openness and visibility	[25,122,123]
Location and orientation	[8,124]
Exterior appearance	[8,124]
Privacy	[28,29,125]
Family service and convenience space	[21,98]
Communication and information	[96,97,126]
Socio-cultural support	[93,95,98]
Medical and administrative staff relationship	[8,124,126]

**Table 2:** Survey respondents’ personal and professional characteristics.

Variable		*n*	%
Sex	Male	131	72.0
	Female	51	28.0
Age (years)	20s	35	19.2
	30s	44	24.2
	40s	74	40.7
	50s or older	29	15.9
Education	Bachelor	87	47.8
	Graduate	79	43.4
	Doctorial	16	8.8
Experience with hospital design (years)	<1	95	52.2
	1–5	34	18.7
	6–10	25	13.7
	>10	28	15.4
Architect licensure ship	Yes	62	34.1
	No	120	65.9
Field of specialty	Healthcare and welfare	69	37.9
	Commercial and cultural	41	22.5
	Office/workspace	13	7.1
	Housing and urban planning	39	21.4
	Educational, etc.	20	11.0
Total		182	100.0

**Table 3:** Principle component analysis and varimax rotation of the important therapeutic environmental criteria (factors and items).

Factor and Items	Factor Loading	Eigenvalue	% Variance	Cronbach’s α
Factor 1: Management		2.700	10.801	0.761
Hygiene	0.732			
Safety	0.708			
Maintenance	0.704			
Factor 2: Interior design		2.628	10.513	0.776
Color composition	0.814			
Coordinated art and image	0.765			
Furniture layout	0.641			
Factor 3: Spatial quality		2.571	10.285	0.765
Openness and visibility	0.745			
Location and orientation	0.657			
Wayfinding	0.653			
Accessibility	0.587			
Spatial privacy	0.476			
Exterior appearance	0.465			
Factor 4: Service		2.285	9.141	0.754
Medical staff’s service	0.832			
Administration staff’s service	0.825			
Family service facility	0.508			
Factor 5: Nature and rest		2.085	8.339	0.712
Outdoor view	0.767			
Natural daylight	0.674			
Green space	0.609			
Rest and recreational space	0.572			
Factor 6: Ambient indoor comfort		2.046	8.183	0.679
Illumination	0.627			
Thermal comfort	0.612			
Air quality	0.588			
Indoor noise	0.508			
Factor 7: Social program and space		1.914	7.655	0.663
Health information program and space	0.789			
Socio-cultural program and space	0.693			
Total variance explained: 64.917%				
Bartlett’s test: chi square = 1783.088, df = 300 (*p* < 0.001), Kaiser–Meyer–Olkin = 0.835				

Note: Two items did not load onto any factor in the factor analysis: ventilation and provision of convenient facility (e.g., hospital store, cafeteria, etc.).

**Table 4:** Mean and standard deviation of important therapeutic environmental criteria (factors and items).

Factors and Items	Mean	Standard Deviation
Factor 1: Management	4.52	0.573
Hygiene	4.65	0.620
Safety	4.51	0.763
Maintenance	4.42	0.698
Factor 2: Interior design	3.59	0.699
Color composition	3.72	0.850
Coordinated art and image	3.43	0.900
Furniture layout	3.63	0.767
Factor 3: Spatial quality	3.77	0.548
Openness and visibility	3.87	0.746
Location and orientation	3.82	0.829
Wayfinding	3.79	0.782
Accessibility	3.80	0.852
Spatial privacy	3.96	0.761
Exterior appearance	3.37	0.868
Factor 4: Service	4.15	0.606
Medical staff’s service	4.42	0.691
Administration staff’s service	4.18	0.762
Family service facility	3.84	0.767
Factor 5: Nature and rest	4.04	0.556
Outdoor view	3.82	0.797
Natural daylight	4.54	0.618
Green space	3.58	0.855
Rest and recreational space	4.21	0.746
Factor 6: Ambient indoor comfort	4.26	0.501
Illumination	4.18	0.677
Thermal comfort	4.37	0.667
Air quality	4.66	0.597
Indoor noise	3.84	0.844
Factor 7: Social program and space	3.39	0.738
Health information program and space	3.23	0.855
Socio-cultural program and space	3.54	0.851

Note: 1: *least important*; 2: *not so important*; 3: *neutral*; 4: *important*; 5: *very important* (*n* = 182).

**Table 5:** Management factors in relation to the respondents’ personal and professional characteristics.

Variable		*f*(*n*)	Mean	Standard Deviation	t/F	*p*
Sex	Male	131	4.45	0.612	3.228	0.002
	Female	51	4.71	0.409		

**Table 6:** Interior design factor in relation to the respondents’ personal and professional characteristics.

Variable		*f*(*n*)	Mean	Standard Deviation	t/F	*p*
Age (years)	20s	35	3.37 a	0.753	2.683	0.048
	30s	44	3.55 ab	0.705		
	40s	74	3.63 ab	0.648		
	50s or older	29	3.85 b	0.688		

a < b: Duncan’s post-verification.

**Table 7:** Spatial quality factor in relation to the respondents’ personal and professional characteristics.

Variable		f(*n*)	Mean	Standard Deviation	t/F	*p*
Sex	Male	131	3.71	0.590	2.608	0.010
	Female	51	3.91	0.391		

**Table 8:** Interview participants’ personal and professional characteristics.

Variables		*n*	%
Sex	Female	8	53.3
	Male	7	46.7
Age (years)	30s	10	66.7
	40s	3	20.0
	50s or older	2	13.3
Education	Bachelor	7	46.7
	Graduate	8	53.3
	Doctorial	0	0.0
Experience in hospital design project management (years)	≤5	2	13.3
	6–10	4	26.7
	11–15	4	26.7
	>15	5	33.3
Architectural licensure ship	Yes	8	53.3
	No	7	46.7
Size of firm (persons)	≤100	4	26.7
	101–300	1	6.7
	301–600	3	20.0
	>600	7	46.7
	Total	15	100.0

## Data Availability

All the data are presented in this study.

## Appendix A

**Table A1:** Participants’ personal priority and firms’ priority in design implementation of the therapeutic environmental variables.

	Factors and Items	Personal		Firm	
		*n*	%	*n*	%
Factor 1: Management	Hygiene	4	26.7	5	33.3
	Safety	3	20.0	3	20.0
	Maintenance	2	13.3	1	6.7
Factor 2: Interior design	Color composition	1	6.7	1	6.7
	Coordinated art and image	0	0.0	0	0.0
	Furniture layout	2	13.3	0	0.0
Factor 3: Spatial quality	Openness and visibility	3	20.0	3	20.0
	Location and orientation	1	6.7	5	33.3
	Wayfinding	3	20.0	4	26.7
	Accessibility	1	6.7	6	40.0
	Spatial privacy	6	40.0	3	20.0
	Exterior appearance	0	0.0	6	40.0
Factor 4: Service	Medical staff’s service	2	13.3	0	0.0
	Administration staff’s service	2	13.3	0	0.0
	Family service facility	1	6.7	0	0.0
Factor 5: Nature and rest	Outdoor view	3	20.0	4	26.7
	Natural daylight	5	33.3	6	40.0
	Green space	2	13.3	2	13.3
	Rest and recreational space	4	26.7	2	13.3
Factor 6: Ambient indoor comfort	Illumination	3	20.0	3	20.0
	Thermal comfort	0	0.0	2	13.3
	Air quality	4	26.7	5	33.3
	Indoor noise	1	6.7	1	6.7
Factor 7: Social program and space	Health information program and space	0	0.0	0	0.0
	Socio-cultural program and space	1	6.7	1	6.7

**Table A2:** Frequency of applied variables in design implementation for therapeutic hospital environment.

	Factors and Items	More Frequent		Less Frequent	
		*n*	%	*n*	%
Factor 1: Management	Hygiene	3	20.0	3	20.0
	Safety	5	33.3	0	0.0
	Maintenance	0	0.0	1	6.7
Factor 2: Interior design	Color composition	0	0.0	3	20.0
	Coordinated art and image	0	0.0	3	20.0
	Furniture layout	0	0.0	3	20.0
Factor 3: Spatial quality	Openness and visibility	5	33.3	6	40.0
	Location and orientation	4	26.7	0	0.0
	Wayfinding	10	66.7	1	6.7
	Accessibility	4	26.7	0	0.0
	Spatial privacy	1	6.7	2	13.3
	Exterior appearance	4	26.7	1	6.7
Factor 4: Service	Medical staff’s service	0	0.0	2	13.3
	Administration staff’s service	0	0.0	2	13.3
	Family service facility	1	6.7	6	40.0
Factor 5: Nature and rest	Outdoor view	4	26.7	5	33.3
	Natural daylight	7	46.7	1	6.7
	Green space	5	33.3	5	33.3
	Rest and recreational space	6	40.0	4	26.7
Factor 6: Ambient indoor comfort	Illumination	1	6.7	2	13.3
	Thermal comfort	0	0.0	3	20.0
	Air quality	2	13.3	3	20.0
	Indoor noise	0	0.0	1	6.7
Factor 7: Social program and space	Health information program and space	0	0.0	1	6.7
	Socio-cultural program and space	2	13.3	2	13.3
